# Use of Clamp-Like Devices to Fix Bifrontal Basal Craniotomies: Three Case Reports

**DOI:** 10.7759/cureus.52417

**Published:** 2024-01-17

**Authors:** Álvaro Gomez de la Riva

**Affiliations:** 1 Department of Neurosurgery, Hospital Universitario La Paz, Madrid, ESP; 2 Department of Medicine, Universidad Europea de Madrid, Madrid, ESP

**Keywords:** frontal bone, neurosurgical procedures, case report, cranial loop, craniotomy

## Abstract

The three case reports presented here provide clinical evidence of a configuration to easily and successfully fix bifrontal basal craniotomies with a clamp-like fixation device (Cranial LOOP™, NEOS Surgery, Barcelona, Spain): A 72-year-old woman undergoing resection of a meningioma at the sphenoidal level, a 43-year-old man undergoing clipping of an anterior communicating artery aneurism, and a 55-year-old woman undergoing macroscopical resection of a meningioma on the olfactory groove had their surgery performed through a bifrontal basal craniotomy, and, once the intervention was completed, the fixation of the cranial flap was performed using Cranial LOOP™, with a configuration consisting of two Cranial LOOP™ L at the basal lateral level and two additional products at the posterior frontal level. The result of this fixation was very satisfactory with no settling or artifacts during the follow-up. These cases show that Cranial LOOP™ can be used to fixate bifrontal basal craniotomies.

## Introduction

Basal bifrontal craniotomies are a surgical procedure in which the frontal part of the skull is removed to access the frontal lobe and the frontal skull base. In some of these cases, the craniotomy must be as basal as possible.

When basal bifrontal craniotomies have to be closed again with the extracted bone flap, plates and screws are the most common method used, as this is the gold standard in cranial fixation. However, this method presents disadvantages, such as artifacts in radiological images and potential complications such as bone resorption, bone flap depression, or skin erosion [1].

An alternative to the use of plates and screws as a fixation system is the use of clamps. Cranial LOOP™ (NEOS Surgery, Barcelona, Spain) a clamp-like polymer-based bone flap fixation device, has been used for more than a decade around the world [2]. Cranial LOOP™ devices consist of two polyether-ether-ketone (PEEK-Optima™, Invibio Ltd., Lancashire, UK) platforms linked by two adjustable ties of the same material. This device fixates the bone flaps based on its clamp principle: one of the platforms is placed below the bone and the other one above. By pulling on the handle and pressing gently on the applier of the device, the two platforms are tightened together. The main advantages of Cranial LOOP™ over other bone flap fixations are the following: (a) it ensures a stable fixation with a very simple technique, (b) it requires no other surgical instrument to perform this fixation, (c) PEEK-Optima™ is not radiopaque, which ensures artifact-free radiological images [3], and (d) PEEK-Optima™ has a lower impact on the radiotherapy dose applied in areas nearby an implant than titanium, reducing the need for corrections when performing radiotherapy in the area below the craniotomy [4]. Cranial LOOP™ comes in three different sizes according to the diameter of the platforms: 12 mm (regular size) and 16 mm (L size) only for bone flap fixation and 22 mm (XL size) to cover burr holes in addition to fixing the bone flap. Its implantation method is explained in the instructions for use provided by the manufacturer [5].

In addition, it has been reported that Cranial LOOP™ offers excellent cosmetic results [3], which makes it a particularly optimal system to close frontal craniotomies as it avoids any bulges or cosmetic disturbances that have been commonly reported for the use of plates and screws. However, according to its instructions for use, Cranial LOOP™ should not be used on the base of the skull. Thus, the use of Cranial LOOP™ in bifrontal craniotomies has been limited because they cannot be implanted on the base of the skull facets of the bone flap, requiring their use in combination with other fixation systems. This is the reason why most surgeons prefer the use of titanium plates and screws in these cases.

The cases presented in this article required a bifrontal basal craniotomy, and once the intervention was finished, the bone flaps were fixated using Cranial LOOP™ only. The author makes use of a specific configuration and distribution of devices, overcoming their limitations by avoiding the base of the skull facet. This allows an easy and satisfactory fixation of the bone flap.

## Case presentation

Case 1

**Figure 1:**
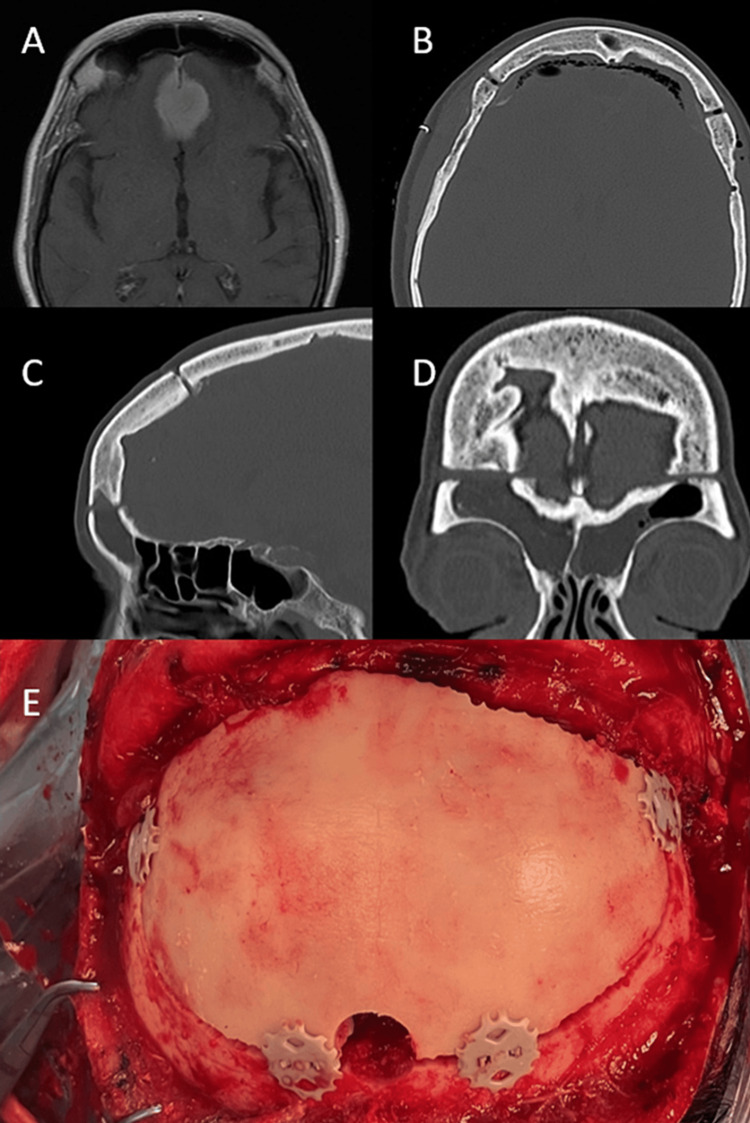
A 72-year-old woman undergoing olfactory groove meningioma resection (A) Pre-operative MRI image: axial view weighted in T1 with gadolinium showing a frontal basal meningioma. (B) Twenty-four-hour post-operative CT scan image: axial view of the bone. (C) Five-month post-operative CT scan image: sagittal reconstruction view of the bone. D) Five-month post-operative CT scan image: coronal reconstruction view of the bone. (E) Surgical view after tumor resection and fixation of the bone flap with Cranial LOOP™.

A White 72-year-old woman with a history of anosmia for 1.5 years presented difficulty in pronouncing some phonemes and temporary dysarthria during the previous week. No alterations were detected during the neurological assessment. A meningioma at the sphenoidal level was observed at the CT scan and the MR was partially calcified, with associated bifrontal vasogenic edema (Figure 1A). The patient underwent macroscopic resection of the tumor through a bifrontal basal craniotomy. Given the extensive edema caused by the meningioma, we chose this surgical approach to have a larger surgical field while avoiding any damage caused by the retraction of the frontal lobes. The bone flap was fixated using two Cranial LOOP™ L at the lateral basal level close to the pterion and two regular-size Cranial LOOP™ at the posterior frontal level (Figure 1E). The patient progressed without any complications. CT scans were performed immediately after surgery (Figure 1B) and at a five-month follow-up visit (Figures 1C-1D), showing correct positioning of the bone flap without settling or artifacts. The patient was satisfied with the aesthetic result and reported no pain.

Case 2

**Figure 2:**
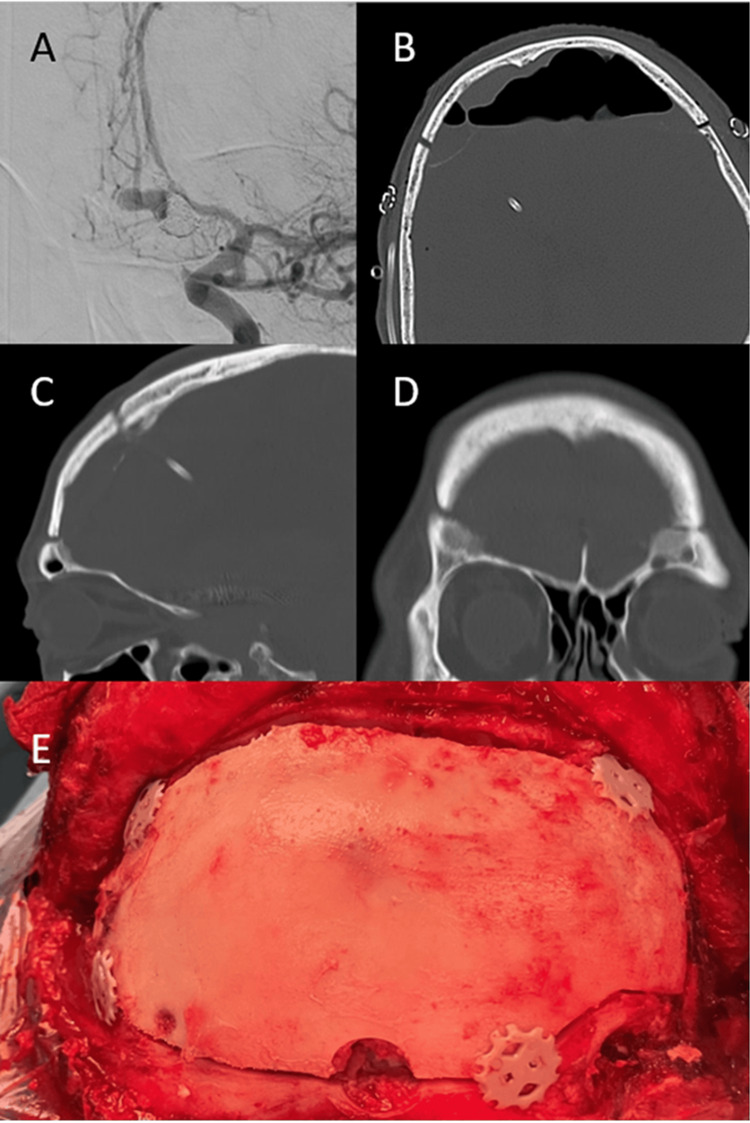
A 43-year-old man undergoing clipping of an anterior communicating artery aneurism (A) Pre-operative arteriography image: anteroposterior view showing the rechannelled anterior communicating artery aneurism. (B) Twenty-four-hour post-operative CT scan image: axial view of the bone. (C) Seven-month post-operative CT scan image: sagittal reconstruction view of the bone. (D) Seven-month post-operative CT scan image: coronal reconstruction view of the bone. (E) Surgical view after microsurgical clipping and fixation of the bone flap with Cranial LOOP™.

A White 43-year-old man with a history of subarachnoid hemorrhage underwent clipping of the anterior communicating artery aneurism via interhemispheric approach. The subarachnoid hemorrhage was caused by a rupture of the anterior communicating artery. The aneurysm was treated nine months prior to surgery using coils and a CSF derivation valve for the hydrocephalus. Later on, the patient had a manifest aneurysmal recanalization (Figure 2A). No alterations were detected during the neurological assessment. Anterior communicating artery aneurism clipping was performed through a bifrontal basal craniotomy. Given that the patient had a complex, recanalized aneurysm with two episodes of subarachnoid hemorrhage, we chose this surgical approach to obtain a wide visualization of the aneurysm and both A2 arteries with less retraction of the frontal lobes. The bone flap was fixated using two Cranial LOOP™ L at the basal lateral level close to the pterion and two regular-size Cranial LOOP™ at the posterior frontal level (Figure 2E). The Cranial LOOP™ placed on the right side of the flap was placed more medially to avoid the LCR derivation valve. The patient progressed without any complications. CT scans were performed immediately after surgery (Figure 2B) and at a seven-month follow-up visit (Figures 2C-2D), showing correct positioning of the bone flap without settling or artifacts. The patient was satisfied with the aesthetic result and reported no pain.

Case 3

**Figure 3:**
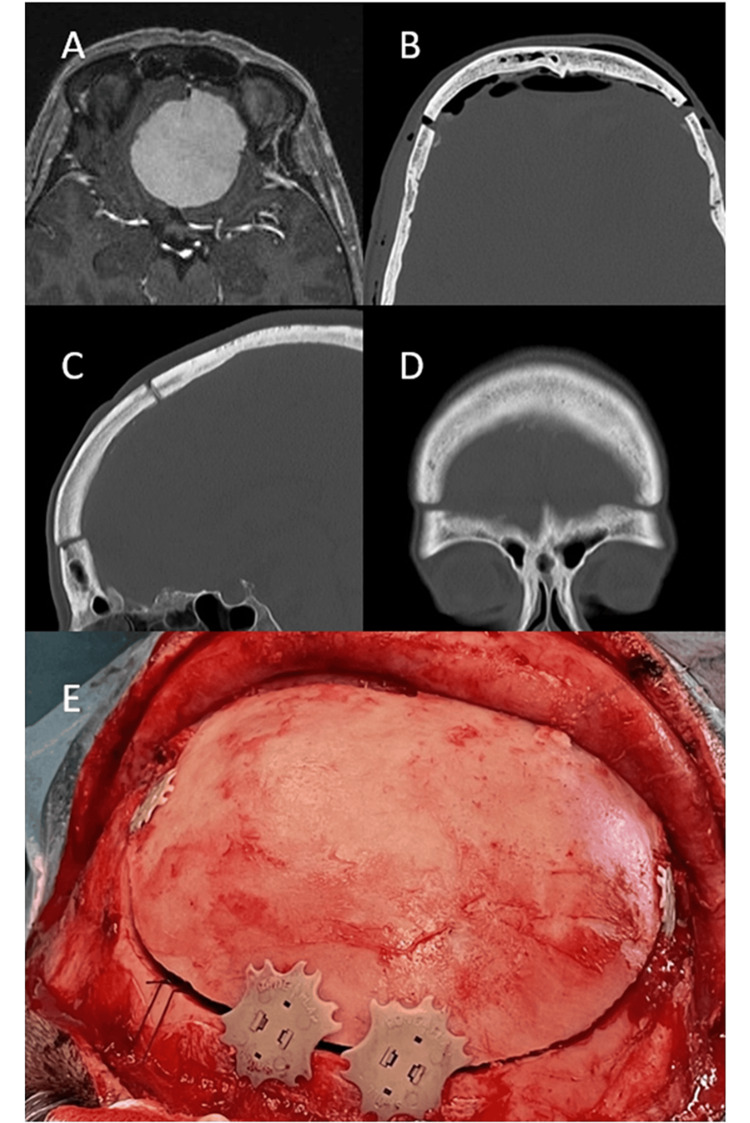
A 55-year-old woman undergoing resection of a meningioma on the olfactory groove (A) Pre-operative MRI image: axial view weighted in T1 with gadolinium showing a frontal basal meningioma. (B) Twenty-four-hour post-operative CT scan image: axial view of the bone. (C) Four-month post-operative CT scan image: sagittal reconstruction view of the bone. (D) Four-month post-operative CT scan image: coronal reconstruction view of the bone. (E) Surgical view after tumor resection and fixation of the bone flap with Cranial LOOP™.

A White 55-year-old woman with symptoms of upper limb paraesthesia was initially diagnosed with multiple sclerosis upon cervical MRI findings compatible with sclerotic plaques. However, further study revealed a 5-cm meningioma on the olfactory groove with edema around the lesion (Figure 3A). After this discovery, the patient reported anosmia after the COVID-19 infection with little recovery. The patient underwent a complete macroscopical resection of the meningioma with tumoral base coagulation through a bifrontal basal craniotomy. A frontal periosteal pedicled flap was placed over the anterior cranial fossa to prevent a potential cerebrospinal fluid fistula. Similar to case 1, this case presented extensive edema and high tumor volume; this is why we chose this surgical approach to have a larger surgical field while avoiding any damage caused by the retraction of the frontal lobes. The cranial flap was fixated with two Cranial LOOP™ L at the basal lateral level close to the pterion and two Cranial LOOP™ XL at the posterior frontal level, covering the burr holes performed on both sides of the superior longitudinal sinus (Figure 3E). The patient progressed without complications. CT scans were performed immediately after surgery (Figure 3B) and at a four-month follow-up visit (Figures 3C-3D), showing correct positioning of the bone flap without settling or artifacts. The patient was very satisfied with the aesthetic result and reported no pain.

## Discussion

This article provides details of three neurosurgical procedures involving bifrontal basal craniotomy and the method used to fixate the bone flap afterward. The main strength of these cases is that they show high reproducibility of the technique, with minor changes to adapt to each case, as well as consistency in the results obtained with this technique: in all cases, the bone flap was correctly positioned and had no settling during the follow-up. With the placement of four Cranial LOOP™ devices on four distal points, a stable cranial closure is achieved. Another advantage of the lateral placement of Cranial LOOP™ is that it leaves the area of the anterior frontal base free of fixation products, thus allowing the introduction of a free flap of periosteum to cover the anterior cranial fossa to prevent a potential cerebrospinal fluid fistula [6].

It is worth noting that this method would not be adequate when an orbital osteotomy is performed because, in these cases, the bone reconstruction can only be performed with plates and screws or using heterologous bone flaps [7]. Another limitation of its use is supraciliary mini-craniotomies, in which the placement of Cranial LOOP™ is technically more difficult due to the small size of the bone flap.

The method used in this article’s cases proposes an interesting alternative that tackles most of the abovementioned issues. Cranial LOOP™ is generally praised for its ease of use, avoiding the use of complex instrument kits that require sterilization [2,3]. The risks of cranial fixation complications such as bone resorptions, skin erosion, or bone flap depression are significantly reduced when using Cranial LOOP™ thanks to the design of this device. On one side, contrary to the fixation of plates to the bone with screws, the clamp-like system does not require implantation on the bone; thus, there is no risk of bone resorption. On the other side, this system provides support underneath the bone flap with its lower platform, thus reducing the risk of bone flap depression. It is also worth noting that Cranial LOOP™ material (PEEK), unlike titanium, does not significantly modify the radiation dose received by the tissues near the implant in radiotherapy [4,8]. Aesthetically, it has good results, and the XL size manages to cover burr holes very efficiently, while it also contributes to the fixation of the bone flap when the superior longitudinal sinus is not involved.

There are other devices available based on the clamp-like system, such as titanium clamps [9] and another PEEK clamp system [10]. However, the titanium clamps still present CT and MRI artifacts, and the PEEK clamp system, although very similar, requires a specific tool to cut the excess parts from implantation and the assembly of four parts, while Cranial LOOP™ is composed of a single piece with a handle that makes implantation easier and instrument-free.

Despite the great reproducibility shown in these cases, the main limitation of this study is the low number of cases presented. However, the aim of this study was not to demonstrate the general safety and performance of Cranial LOOP™, which had been previously studied [2,3,9], but to demonstrate its use in a cranial area that had previously proven difficult for clamp-like systems.

## Conclusions

The takeaway lesson from these cases is that Cranial LOOP™ can be used to easily fixate the bone flap of bifrontal basal craniotomies, and the best way to perform this fixation is with two Cranial LOOP™ at the basal lateral level and two Cranial LOOP™ at the posterior frontal level. Despite the difficult skull anatomy of bifrontal craniotomies, the result of this fixation with clamp-like devices was satisfactory, with no settling or aesthetic defects during the follow-up.

## References

[1] Yeap MC, Tu PH, Liu ZH (2019). Long-term complications of cranioplasty using stored autologous bone graft, three-dimensional polymethyl methacrylate, or titanium mesh after decompressive craniectomy: a single-center experience after 596 procedures. World Neurosurg.

[2] Van Loock K, Menovsky T, Kamerling N, De Ridder D (2011). Cranial bone flap fixation using a new device (Cranial LoopTM). Minim Invasive Neurosurg.

[3] Asencio-Cortés C, Salgado-López L, Muñoz-Hernandez F, de Quintana-Schmidt C, Rodríguez-Rodríguez R, Álvarez-Holzapfel MJ, Conesa G (2019). Long-term safety and performance of a polymeric clamplike cranial fixation system. World Neurosurg.

[4] Katsifis GA, McKenzie DR, Suchowerska N (2022). Monte Carlo calculations of radiotherapy dose distributions within and around orthopaedic implants. Phys Imaging Radiat Oncol.

[5] NEOS Surgery S.L (2018). Cranial LOOP™: reinventing cranial fixations. https://neosurgery.com/product/cranial-loop/.

[6] Price JC, Loury M, Carson B, Johns ME (1988). The pericranial flap for reconstruction of anterior skull base defects. Laryngoscope.

[7] Bečulić H, Spahić D, Begagić E (2023). Breaking barriers in cranioplasty: 3D printing in low and middle-income settings-insights from Zenica, Bosnia and Herzegovina. Medicina (Kaunas).

[8] Nevelsky A, Borzov E, Daniel S, Bar-Deroma R (2017). Perturbation effects of the carbon fiber-PEEK screws on radiotherapy dose distribution. J Appl Clin Med Phys.

[9] Aboulfetouh I, Younes YW (2019). Cranial bone flap fixation: comparison of titanium-based device (skull fix) and peek-based device (cranial loop): technical report. Med J Cairo Univ.

[10] Piccirilli M, Spena G, Marchese E, Tropeano MP, Santoro A (2021). A new device for bone cranial flap fixation: technical note and surgical remarks. A multicentric experience. Surg Neurol Int.

